# Identification of microRNA-21 target genes associated with hair follicle development in sheep

**DOI:** 10.7717/peerj.7167

**Published:** 2019-06-27

**Authors:** Bo Zhai, Lichun Zhang, Chunxin Wang, Zhuo Zhao, Mingxin Zhang, Xu Li

**Affiliations:** Jilin Academy of Agricultural Science, Branch of Animal Husbandry, Gongzhuling, China

**Keywords:** microRNA-21, Sheep, Hair follicles

## Abstract

**Aim:**

The target molecule regulatory function of microRNA-21 (miR-21) in multiple signalling pathways has become a main focus of genetic and pharmacological regulatory studies of various diseases. The identification of target genes for miRNA-21 in the development of hair follicles can provide new research pathways for the regulation of cell development.

**Methods:**

In the present study, eight six-month-old ewes from Super Merino (SM) and Small Tailed Han (STH) sheep breeds were selected. Target prediction and dual-luciferase wild-type and mutant vectors were used to identify the target genes of miR-21. Quantitative reverse transcription polymerase chain reaction (RT-qPCR) and bioinformatics analysis were conducted to analyze the effects of miR-21.

**Results:**

The results show that the expressions of *CNKSR2*, *KLF3* and *TNPO1* were downregulated by miRNA-21 at rates of 36%, 26% and 48%, respectively. Moreover, there was a significant negative correlation between the expression of miR-21 and the three target genes in sheep with two extreme phenotypes. The expression of microRNA-21in October was significantly lower than that in January and February; while the expression of* CNKSR2*, *KLF3* and *TNPO1* in October was higher than that in January and February. Conclusions: These results suggest that *CNKSR2*, *KLF3* and *TNPO1* are three newly discovered target genes of miR-21 and might be involved in the effects of miR-21 on hair follicle development.

## Introduction

The skin is the largest organ system covering the surface of animals’ bodies and is divided into the epidermis, dermis and subcutaneous tissue. Hair follicles (HF) are a complex morphological and structural appendage of the skin that controls the growth of hair and whose most prominent characteristic is regeneration. HF consist of epithelial and dermal tissues (Alonso & Fuchs, 2006). The development process of hair follicles comprises a series of activities, such as proliferation, apoptosis, differentiation, migration and mutual connection between skin epithelial cells and dermal cells. HF can be divided into primary hair follicles and secondary hair follicles. An important characteristic of HFs is periodic growth, and HF usually undergo recurrent phases of growth (anagen), regression (catagen), and resting (telogen) with a defined periodicity (Millar, 2002; Botchkarev & Kishimoto, 2003). During the HF growth phase, stem cells are activated, hair buds extend downwards and interact with the hair papillae, and hair bulb epithelial cells and hair matrix cells continuously divide and proliferate, forming hair bulbs, inner root sheaths and hair shafts. With the growth of hair follicles, the HFs reach deep into the dermis and accessory structures, such as sebaceous glands and piloerection muscles, are also generated. During the retrograde period, the proliferation of dermal papilla cells is decreased, a large number of cells became apoptotic, the hair bulb epithelial tissues shrink, the dermal papillae are exposed, and the hair follicles became shorter overall, forming rod-like hairs. In the rest period, apoptosis is terminated, and the dermal papillae are in a dormant state (Schneider, Schmidt-Ullrich & Paus, 2009).

MicroRNAs (miRNAs) are a class of endogenous genes that encode 22-nucleotide-long, non-coding, single-stranded RNA molecules that affect gene function by post-transcriptional regulation. Researchers have found that a variety of microRNAs are involved in the development of skin HFs (Andl et al., 2006; Liu et al., 2012; Zhu et al., 2010). HFs of the skin were sequenced during the growth, anagen and resting phases of *Ovis aries* and it was found that microRNA may regulate HF growth by modulating target genes in pathways such as MAPK and Wnt (Liu et al., 2013a; Liu et al., 2013b). MicroRNA-214 controls skin and HF development by modulating the activity of the Wnt pathway (Ahmed et al., 2014). miR-24 affects HF morphogenesis by targeting Tcf-3 (Amelio et al., 2013). miR-31 can regulate keratinocyte growth and hair differentiation through the target genes STK40 and LATS2(Luan et al., 2017). miR-200b and miR-196a may be associated with HF growth in transgenic mice by intraepithelial overexpression of DKK1 (Ryan, Olivera-Fernandes & Lavker, 2006). miR-184 can interfere with miR-205 and may inhibit HF growth and promote depression (Yu et al., 2008). miR-18b inhibits TGF- *β*1-mediated differentiation of human HF mesenchymal stem cells into smooth muscle cells by modulating the target gene SMAD2 (Liu et al., 2013a; Liu et al., 2013b). Post-transcriptional regulation of miR-22 may induce HF regression (Yuan et al., 2015). MicroRNA-21 is an important downstream component of BMP signalling in epidermal keratinocytes (Ahmed et al., 2011). At present, these reports mostly screen and identify microRNAs and their predicted gene targets; research addressing the regulatory mechanisms of the development of skin HF has received less attention. The elucidation of the identification and regulation of specific microRNAs has important implications for skin diseases and hair growth.

Here, we explore the applications of bioinformatics and a luciferase reporter assay in vitro to identify the target genes of miR-21. The results of the present study provide a basis for further investigation of miRNA-mediated HF development in sheep. New target genes would provide more pathways for the study of miR-21 in other animals.

## Materials and Methods

**Ethics statement.** This study was carried out in strict accordance to relevant guidelines and regulations by the Ministry of Agriculture of the People’s Republic of China. All experimental protocols were approved by the Laboratory Animal centre of Jilin University (SKXK 2015-0006).

**Animals**. Eight six-month-old ewes from Super Merino (SM) and Small Tailed Han (STH) sheep breeds were selected from Jinlin province. To eliminate environmental effects, the two groups of ewes were housed on the same farm and provisioned with the same complete formula feed and hay. All skin and samples were collected during mid-October when the wool cycle was expected to be in the anagen phase.

### Target prediction

Target prediction of microRNA-21 was performed using the algorithms TargetScan (http://www.targetscan.org/), Pita (http://genie.weizmann.ac.il/pubs/mir07/mir07_dyn_data.html) and miRanda (http://www.microrna.org/microrna/home.do). These algorithms are available from a previous report (Sun et al., 2014). In brief, TargetScan predictions are based on the matching of sites between the seed regions of microRNAs and mRNAs and miRanda predictions are based on the double-stranded free energy and species conservation. Pita predictions are based on target-site accessibility. All the prediction processes were conducted using custom-written executable files that computed the parameters between microRNAs and mRNAs based on the inherent algorithms and the set thresholds. The thresholds for the algorithms were as follows: the scores ≥70 for TargetScan; Δ ΔG ≤-5 for Pita; and seed ≥7 for miRanda. The intersections of the output results of the three algorithms were used as prediction results for the differentially expressed microRNAs (Sun et al., 2016).

### Dual-luciferase reporter vector construction and luciferase reporter assays

The reaction conditions were as follows (landing PCR): 98 °C for 2 min of denaturation; cycling with denaturation at 98 °C for 10 s, annealing every loop drop 1 °C. From 65 °C, extension at 72 °C for 30 s; followed by 10 cycles starting with denaturation again at 98 °C for 10 s, annealing at 60 °C and then extension at 72 °C for 30 s. Finally, 15 cycles were performed, the extension was continued at 72 °C for 3 min after PCR cycles and the reactions were then stored at 4 °C.

PCR primers were designed based on the seed region sequences of candidate target genes (*Ovis aries*) with 3′ UTRs provided by the National Center for Biotechnology Information (NCBI). Primers targeting the gene segments containing the seed sequences were designed with Xho I and Not I restriction enzyme sites incorporated. In addition, mutagenic primers were designed to specifically mutate 7 bases in the seed region of the candidate target genes. The primer sequences are listed in Table 1. Then, candidate target sequences containing wild-type and mutant seed regions were synthesized. The wild-type and mutant sequences were cloned into pmiR-RB-REPORT™ to construct wild-type and mutant reporter plasmids, respectively. The recombinant vector was sent to Guangzhou Ruibo Biotechnology Co., Ltd. for sequencing.

**Table 1:** Primers of targeting the seed region of the candidate target genes.

Name	Sequence(5′–3′)
CNKSR2-W-F	GGCGG**CTCGAG**TACACTGCGAGAGTTGGTAGA
CNKSR2-W-R	AAT**GCGGCCGC**CAAGAAGTAAGGAGAAAGTTAGG
CNKSR2-M-F	ACAGATTGTATTCGAAATGTTTAGAGAATT
CNKSR2-M-R	TAAACATTTCGAATACAATCTGTGAGTCCAATG TAG
KLF3-W -F	GGCGG**CTCGAG**AGTGTTGCACTAATGTGGA
KLF3-W -R	AAT**GCGGCCGC**GAGTAGAAACAGAGAAGGAAC
KLF3-M -F	TATATTTTTATTCGAAATAAGACTGAATGGGTA
KLF3-M -R	GTCTTATTTCGAATAAAAATATATTACACTTTA
TNPO1-W -F	GGCGG**CTCGAG**TAAGATTGGATGAGTTTTATGGAG
TNPO1-W -R	AAT**GCGGCCGC**ACCCTCAAAACAAAAACCAAG
TNPO1-M -F	AATTTTTGTATTCGAGCTGAATTTTAAAGAGAG
TNPO1-M -R	AATTCAGCTCGAATACAAAAATTAAAACCGTAT

**Notes.**

Bold letter is the mutant sequence.

F forward primer R reverse primer W wild type M mutant type

MiR-21 mimics (5 pmol) or negative control (NC 5 pmol) miR (Shanghai GenePharma Co., Ltd., China) of sheep were cotransfected with 2 ng of the candidate target gene-containing wild type, mutant or empty pmiR-RB-REPORT™ vector (negative control) into the 293T human renal epithelial cell line. The sequences are listed in Table 2. After culture for 48 h, Renilla luciferase and firefly luciferase activities were measured using a chemiluminescence reader.

**Table 2:** MiR-21 mimics / NC sequence.

Name	Sequence(5′–3′)
MiR-21-mimics-F	UAGCUUAUCAGACUGAUGUUGAC
MiR-21- mimics-R	CAACAUCAGUCUGAUAAGCUAUU
NC-F	UUCUCCGAACGUGUCACGUTT
NC-R	ACGUGACACGUUCGGAGAATT

**Notes.**

NC control group F forward primer R reverse primer

### RNA extraction and quantitative real-time reverse transcription polymerase chain reaction (RT-qPCR)

In October, January and February, to capture the different stages in hair follicle growth, four sheep were randomly selected. Total RNA from skin collected from the sides of the sheep was extracted using Trizol reagent (Invitrogen, USA) in accordance with the instruction manual provided by the manufacturer. Reverse transcription of miR-21 was conducted using the PrimeScript™ RT reagent Kit (TaKaRa, Japan). Reverse transcription reactions were prepared as follows: 2 µl of 5x PrimeScript Buffer, 0.5 µl of PrimeScript RT Enzyme Mix I, miR-21 RT primer (10 µM), 0.5 µl of U6 RT mix, and RNase-free ddH2O up to 10.0 µl. Reactions were performed with the following conditions: 37 °C, 15 min; 42 °C, 2 min; and 85 °C, 5 s. The reaction products were stored in a −80 °C freezer.

RT-qPCR was performed using SYBR Premier Dimer EraserTM (TaKaRa, Japan) and cDNA as the template according to the manufacturer’s instructions. The reaction mixes were prepared as follows: 0.5 µl of upstream primer (10 pmol/L), 0.5 µl of downstream primer (10 pmol/L), 8.0 µl of ddH2O, 1 µl of template and 10 µl of SYBR Premix Ex Taq II (2x). Reactions were performed with the following conditions: 95 °C for 30 s; and 40 cycles of 95 °C for 5 s followed by 60 °C for 30 s. The U6 small nuclear RNA (snRNA) was used as an internal control for miR-21, and glyceraldehyde-3-phosphate dehydrogenase (GAPDH) was used as an internal control for other genes. Experimental data were analysed using a relative quantification method (2^−△△*CT*^). All reactions were performed in triplicate. The RT-qPCR primers are described in detail in Table 3.

**Table 3:** Primers used for RT-qPCR analysis.

Name	Sequence(5′–3′)
MiR-21-RT	GTCGTATCCAGTGCGTGTCGTGGAGTCGG CAATTGCACTGGATACGAC
MiR-21-F	GGGTAGCTTATCAGACTGAT
MiR-21-R	CAGTGCGTGTCGTGGAGT
U6-F	CTCGCTTCGGCAGCACA
U6-R	AACGCTTCACGAATTTGCGT
CNKSR2-F	TTAGCTGAGAGCGAGCGGAT
CNKSR2-R	CGCATTCAGACAGCAGTGGT
KLF3-F	GTCATCTACTCCACGCCATTGTCG
KLF3-R	GCTCCGCTTGTTGACTGTGAGG
TNPO1-F	GCTTCCTTCCGTACTGTGAACCTG
TNPO1-R	AATGTTGCCTCCAAGTCCTTCAGC
GAPDH-F	GGTGAAGGTCGGTGTGAACG
GAPDH-R	CTCGCTCCTGGAAGATGGTG

**Notes.**

U6 internal reference F forward primer R reverse primer RT Stem-loop RT primer

### Statistical analysis

All quantitative data and luciferase reporter data are presented as the means ± SDs, and differences were assessed using one-way ANOVA followed by the Bonferroni multiple-comparison test. Values of *p* < 0.01 were considered statistically significant, and all analyses were performed using SPSS 20.0.

## Results

### Prediction of miR-21 target genes

**Figure 1:**
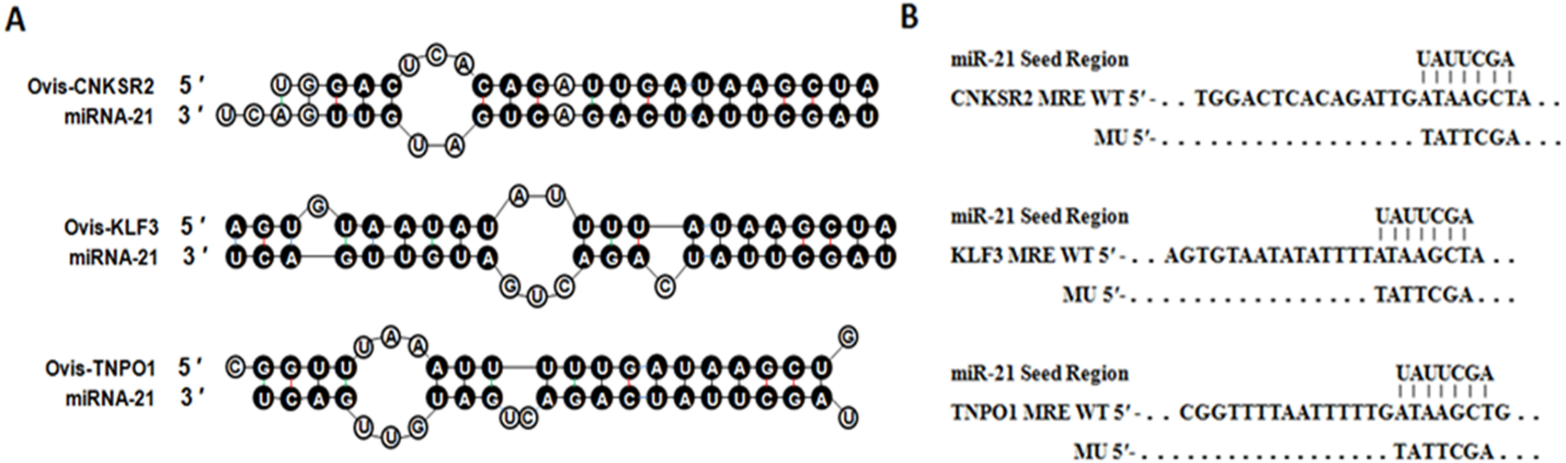
Duplex structure between miR-21 and target genes. (A) Duplex structure between miR-21 and MRE in the *CNKSR2*, *KLF3* and *TNPO1* mRNA predicted by mfold program (http://mfold.rna.albany.edu/?q=mfold). (B) Diagrams of WT or mutant MU constructs of miR-21 MRE in the *CNKSR2*, *KLF3* and *TNPO1* mRNA. Four nucleotides mutations were generated in the *CNKSR2*, *KLF3* and *TNPO1* binding sites to miR-21 seed region using pmiR-RB-REPORT^TM^.

According to the rules of bioinformatics for predicting target genes, miR-21 starts from the second base pair, and the three genes at the 3′ UTR sequence are matched with each other. The number of paired bases is more than seven, as shown in Fig. 1. The combined free energy is −16.7, −10.0 and −14.5 kcal/mol, respectively. Seed sequences are strictly conserved in species such as humans, cattle, sheep, and rats. Therefore, these three genes can be used as candidate target genes for later identification and verification.

### Identification and expression of *CNKSR2* target genes

**Figure 2:**
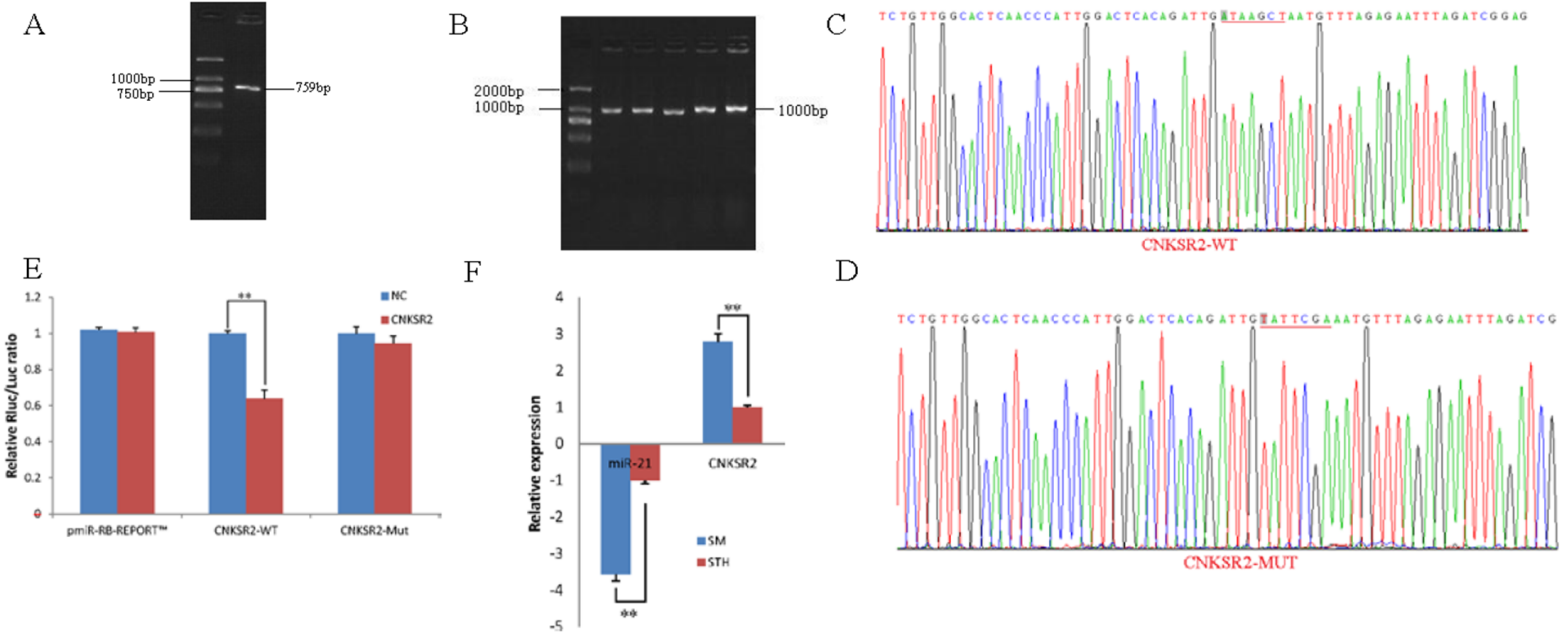
Dual luciferase reporter vector construction of *CNKSR2*. (A) Landing PCR products, M: D2000; the size of the amplification is consistent with the theoretical size. (B) Construction of Vector Plasmids, M: D2000; the size of the amplification is consistent with the theoretical size. (C) Vector plasmid sequencing results. (D) Vector plasmid point mutation sequencing results. (E) Dual luciferase assays with *CNKSR2* MRE wild-type (WT) (pmiR-RB-REPORT^TM^ -WT-CNKSR2) or mutant (Mut) (pmiR-RB-REPORT^TM^ -MUT- CNKSR2) constructs in the presence of miR-21 mimics or NC. (F) The relative mRNA expression levels of miR-21, CNKSR2 between SM sheeps and STH sheeps by RT-qPCR. Data are indicated as the means ± SE derived from triplicate transfectants of three independent experiments. * *p* < 0.05 stands for significant difference; ** *p* < 0.01 stands for extremely significant difference; *p* > 0.05 stands for no significant difference.

To obtain the gene sequences, landing PCR was used to amplify *CNKSR2* (Fig. 2A) and sequences with a length of 759 bp were identified by agarose gel electrophoresis; this length is consistent with the length provided in the nucleic acid database. The size of the 3′ UTR fragment obtained after digestion was confirmed to be the 1,000 bp expected size (Fig. 2B). The forward and reverse sequencing maps showed that the 3′ UTR sequence in the recombinant vector was exactly the same as the length of the PCR product (Fig. 2C) and the length provided in the GenBank database. The CDS sequence of the provided gene was the same, so the reporter recombinant vector was named *CNKSR2*-WT. After a similar procedure, the target sequence ATAAGCT was mutated to TATTCGA (Fig. 2D), and the remaining sequences were not changed. The reporter recombinant vector was named CNKSR2-Mut.

Under normal circumstances, the reporter fluorescence of miRNA mimics a 30% downregulation or more compared with that of the NC controls; after the corresponding binding site is mutated, the inhibition degree of the miRNA mimic reporter fluorescence is significantly reduced or eliminated compared with that of the mutant vector. We can infer that miRNA mimics can regulate reporter gene expression through this site. The dual-luciferase reporter gene expression analysis showed that the oar-miR-21 mimic significantly downregulated the reporter fluorescence of the three wild-type vectors. The reduction of the fluorescence rate of *CNKSR2* was 36%. After the corresponding binding site was mutated, the reporter fluorescence in the mutant vector was significantly restored. Under this experimental model, oar-miR-21 may regulate the expression of the reporter gene through the corresponding 3′ UTR site on *CNKSR2* (Fig. 2E). When the mRNA expression levels of miR-21 and *CNKSR2* in the two extreme phenotypes were negatively correlated, highly significant differences were observed (Fig. 2F).

### Identification and expression of KLF3 target genes

**Figure 3:**
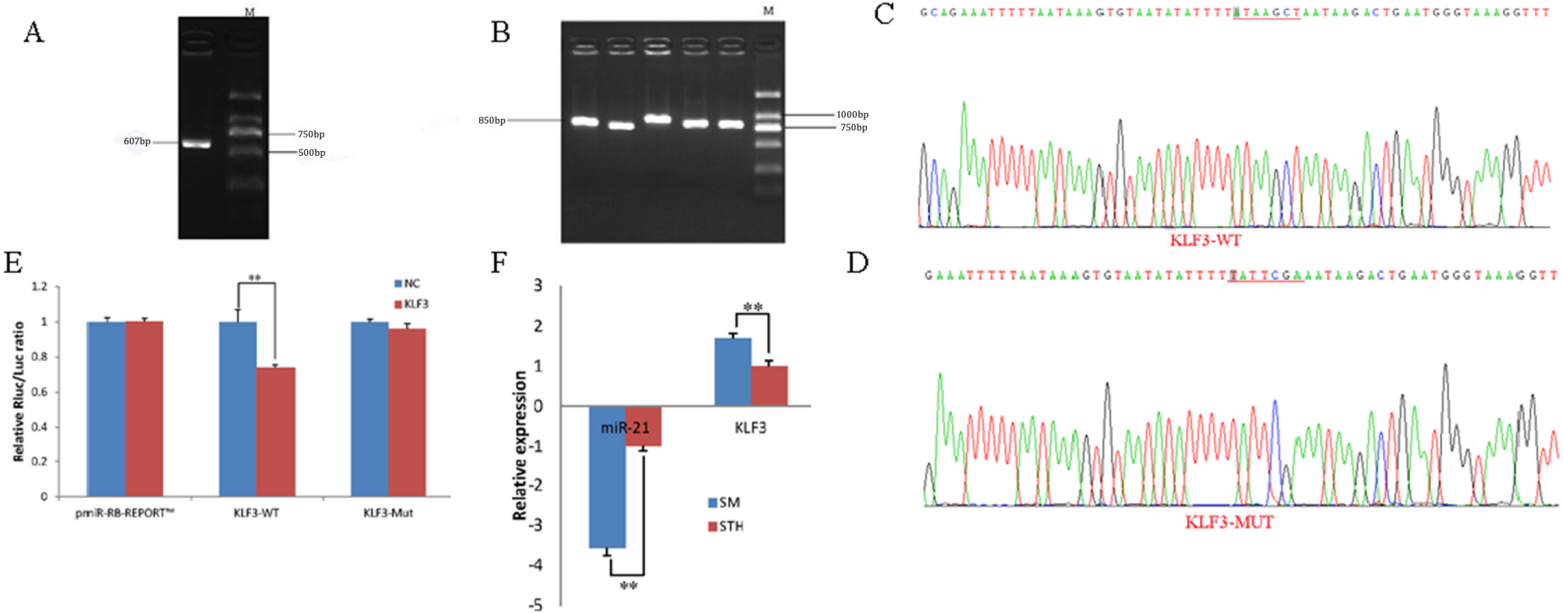
Dual luciferase reporter vector construction of *KLF3*. (A) Landing PCR products, M: D2000; the size of the amplification is consistent with the theoretical size. (B) Construction of Vector Plasmids, M: D2000; the size of the amplification is consistent with the theoretical size. (C) Vector plasmid sequencing results. (D) Vector plasmid point mutation sequencing results. (E) Dual luciferase assays with KLF3 MRE wild-type (WT) (pmiR-RB-REPORT^TM^ -WT- KLF3) or mutant (Mut) (pmiR-RB-REPORT^TM^ -MUT- KLF3) constructs in the presence of miR-21 mimics or NC. (F) The relative mRNA expression levels of miR-21, *KLF3* between SM sheeps and STH sheeps by RT-qPCR. Data are indicated as the means ± SE derived from triplicate transfectants of three independent experiments. *p < 0.05 stands for significant difference; **p < 0.01 stands for extremely significant difference; *p* > 0.05 stands for no significant difference.

To obtain the gene sequences, landing PCR was used to amplify *KLF3* (Fig. 3A). Fragments with a length of 607 bp were identified by agarose gel electrophoresis; this length is consistent with the length provided in the nucleic acid database. The expected size of the 3′ UTR fragment of the 850 bp obtained after digestion was confirmed (Fig. 3B). The forward and reverse sequencing maps showed that the 3′ UTR sequence in the recombinant vector was exactly the same as the length of the PCR product (Fig. 3C) and the length provided in the GenBank database. The CDS sequence of the provided gene was the same, so the reporter recombinant vector was named *KLF3*-WT.

After a similar procedure, the target sequence ATAAGCT was mutated to TATTCGA (Fig. 3D), and the remaining sequences were not changed. The reporter recombinant vector was named *KLF3*-Mut. The reduction of the fluorescence rate of *KLF3* was 26%. After the corresponding binding site was mutated, the reporter fluorescence in the mutant vector was significantly restored. Under this experimental model, oar-miR-21 may regulate the expression of the reporter gene through the corresponding 3′ UTR site on *KLF3*. When the mRNA expression levels of miR-21 and *KLF3* in the two extreme phenotypes were negatively correlated, highly significant differences were observed (Fig. 3F).

### Identification and expression of *TNPO1* target genes

**Figure 4:**
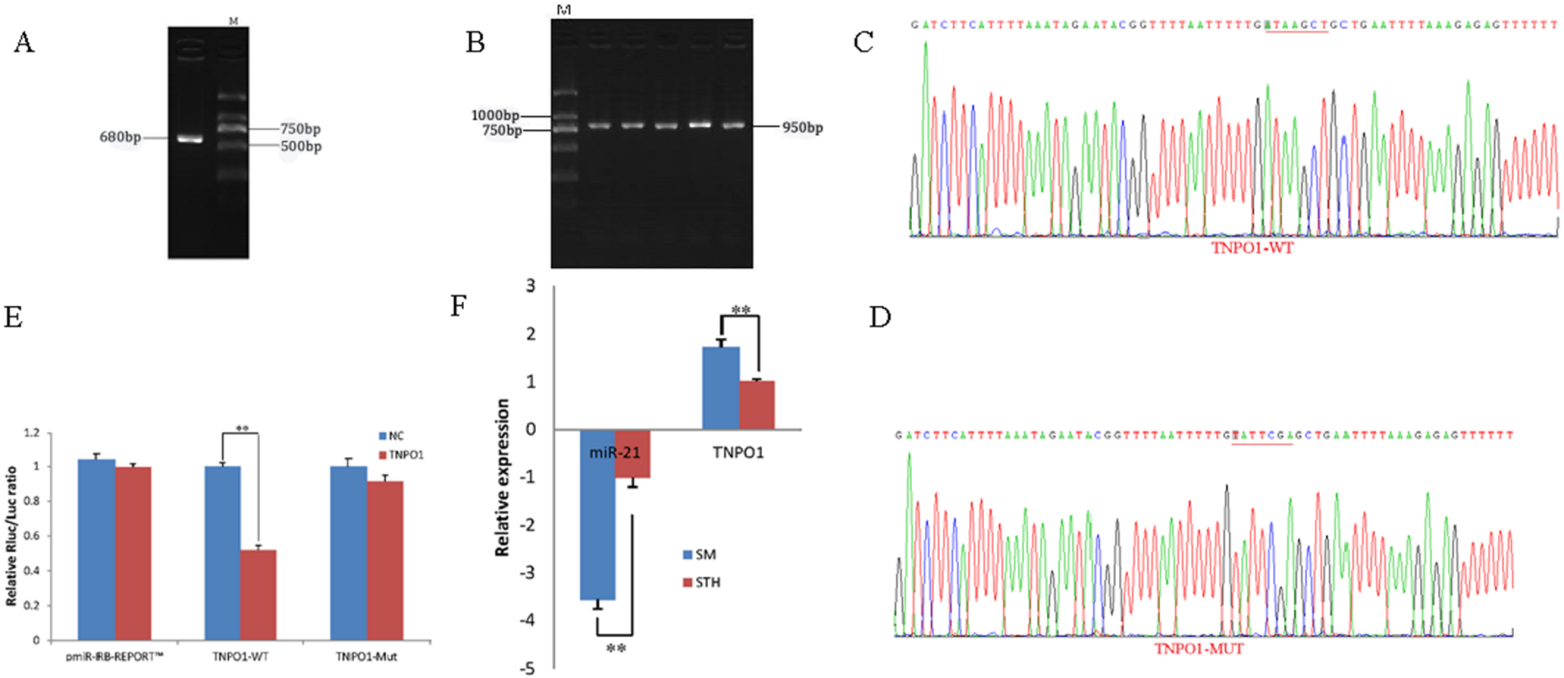
Dual luciferase reporter vector construction of *TNPO1*. (A) Landing PCR products, M: D2000; the size of the amplification is consistent with the theoretical size. (B) Construction of Vector Plasmids, M: D2000; the size of the amplification is consistent with the theoretical size. (C) Vector plasmid sequencing results. (D) Vector plasmid point mutation sequencing results. (E) Dual luciferase assays with *TNPO1* MRE wild-type (WT) (pmiR-RB-REPORT^TM^-WT-TNPO1) or mutant (Mut) (pmiR-RB-REPORT^TM^ -MUT- TNPO1) constructs in the presence of miR-21 mimics or NC. (F) The relative mRNA expression levels of miR-21, TNPO1 between SM sheeps and STH sheeps by RT-qPCR. Data are indicated as the means ± SE derived from triplicate transfectants of three independent experiments. *p < 0.05 stands for significant difference; **p < 0.01 stands for extremely significant difference; *p* > 0.05 stands for no significant difference.

To obtain the gene sequences, landing PCR was used to amplify *TNPO1* (Fig. 4A). Fragments with a length of 680 bp were identified by agarose gel electrophoresis; this length is consistent with the length provided in the nucleic acid database. The expected size of the 3′ UTR fragment of the 950 bp obtained after digestion was confirmed (Fig. 4B). The forward and reverse sequencing maps showed that the 3′ UTR sequence in the recombinant vector was exactly the same as the length of the PCR product (Fig. 4C) and the length provided in the GenBank database. The CDS sequence of the provided gene was the same, so the reporter recombinant vector was named *TNPO1*-WT.

After a similar procedure, the target sequence ATAAGCT was mutated to TATTCGA (Fig. 4D), and the remaining sequences were not changed. The reporter recombinant vector was named *TNPO1*-Mut. The reduction of the fluorescence rate of *TNPO1* was 48%. After the corresponding binding site was mutated, the reporter fluorescence in the mutant vector was significantly restored. Under this experimental model, oar-miR-21 may regulate the expression of the reporter gene through the corresponding 3′ UTR site on *TNPO1*. When the mRNA expression levels of miR-21 and *TNPO1* in the two extreme phenotypes were negatively correlated, highly significant differences were observed (Fig. 4F).

### The expression pattern of miR-21 and three target genes in hair follicle development

**Figure 5:**
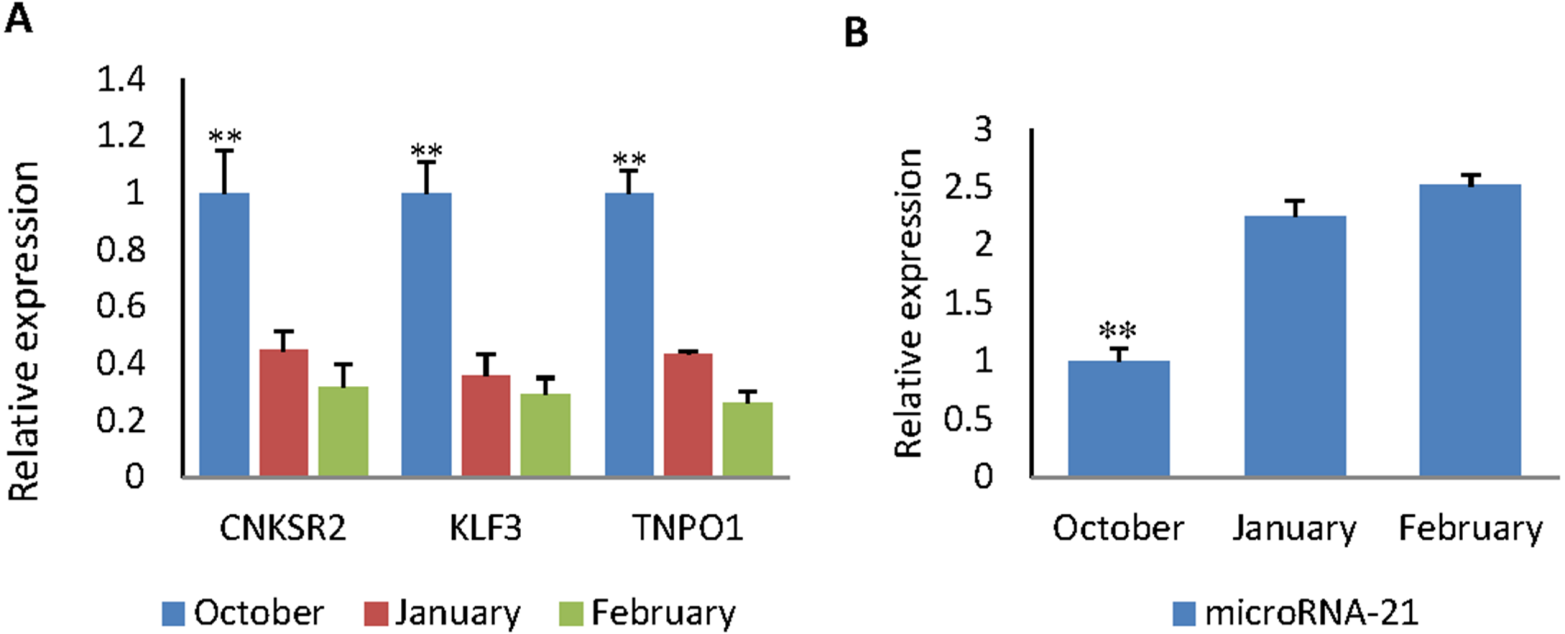
The expression levels of miR-21 and target genes at different wool follicle development stage. October: HF flourishing period ;January: HF degenerative period ;February: HF resting period. (A) The relative mRNA expression levels of target genes. (B) The relative mRNA expression levels of miR-21. Data are indicated as the means ± SE derived from triplicate transfectants of three independent experiments. *p < 0.05 stands for significant difference; **p < 0.01 stands for extremely significant difference; *p* < 0.05 stands for no significant difference.

RT-qPCR was used to obtain the expression pattern, 2^−ΔΔ*Ct*^ method was used to quantify diferential expression level. The expression level of *CNKSR2/KLF3/TNPO1* in October (HF flourishing period) are significantly higher than other months (*p* < 0.01) (Fig. 5A).The expression level of miR-21 in October are significantly decreases compared to other months (*p* < 0.01) (Fig. 5B). The expression level in January (HF degenerative period) and February (HF resting period) have no significant difference (*p* < 0.05).

## Discussion

miRNA plays an important role in the growth and development of animals and exerts a variety of biological functions in various tissues and organs. Studies have found that miRNA-21 can regulate the proliferation and apoptosis of many kinds of cells (Feng et al., 2018; Hou et al., 2018; Zhou et al., 2018). miR-21 inhibits apoptosis in gliomas by inhibiting the expression of caspase 3 and 7 (Chan, Krichevsky & Kosik, 2005; Corsten et al., 2007). In addition to relying on caspases to regulate apoptosis, found that miR-21 can also affect the apoptosis of glioma cells through targeted regulation of the tumour suppressor gene PDCD4 (Chen et al., 2008). The study found that miR-21 can also target negatively regulated genes such as *p53* and *TGF- β* as well as the mitochondrial apoptosis pathway gene for the voltage-dependent anion channel 1 (*VDAC1*) (Papagiannakopoulos, Shapiro & Kosik, 2008). In addition, miR-21 can also inhibit apoptosis by regulating FasL (Liu et al., 2018). Abnormally high expression of miR-21 in breast cancer patients suggests that miR-21 can promote tumour cell growth (Si et al., 2007). miR-21 regulates cellular proliferation, invasion, migration, and apoptosis by targeting *PTEN*, *RECK* and *Bcl-2* in lung squamous carcinoma (Xu et al., 2014). miR-21 also has an inhibitory effect on the apoptosis of non-cancer cells. miR-21 is overexpressed in response to high glucose and protects endothelial cells from apoptosis (Zeng et al., 2013). Antagonists of miRNA-21 can regulate the proliferation and apoptosis of adventitial fibroblasts and myofibroblasts (Wang et al., 2012). miR-21 is the only post-transcriptional regulator that has a highly conserved effect on apoptosis in species.

In the present study, we predicted three target genes of miR-21 by the principles of free energy and base pairing and successfully constructed a dual-luciferase wild-type vector with three genes, CNKSR2, KLF3 and TNPO1. The base sequence ATAAGCT was mutated to TATTCGA, and a double-luciferase mutant vector of three genes was constructed. The successful construction of these two vectors provides the experimental basis for subsequent dual-luciferase reporter assays and functional identification of candidate genes.

To investigate whether miR-21 plays a role in HF development, the expression of miR-21 and its target genes were identified in two sheep varieties with extreme phenotypes and different development stages of HF, indicating that the three genes were regulated by miR-21 expression in the development of HFs. CNK (connector enhancer of kinase suppressor of Ras) is a putative multi-adaptor scaffold protein required in multiple receptor tyrosine kinase pathways, particularly in the RAS-RAF/ mitogen–activated protein kinase (MAPK) cascade, which is involved in conveying several proliferative and differentiative signals to the nucleus (Therrien, Wong & Rubin, 1998). The effect of miR-21 on the target regulation of the *CNKSR2* gene may be mediated by modulating the PI3K-PTEN-AKT-FoxO3a pathway via the scaffold protein *CNKSR2*, which is involved in RAS-dependent signalling pathways. Thus, the proliferation of HF cells and the differentiation of HF stem cells were regulated, and the miR-21-*CNKSR* 2-*MAPK* signalling pathway was formed to regulate HF development. *KLF3* has diverse biological roles, regulating proliferation, differentiation, and apoptosis in many tissues throughout development. *KLF3* could inhibit cellular growth and suppress transformation mediated by oncogenic KRAS (V-Ki-ras2 Kirsten rat sarcoma viral oncogene homologue) and increase apoptosis in cancer cells (Fernandez-zapico, Lomberk & Tsuji, 2011). miR-204-5p regulates adipocyte differentiation by negatively regulating *KLF3*, a negative regulator of lipogenesis (Du, Zhang & Gan, 2018). In the case that *KLF3* plays a significant role in cell proliferation and apoptosis, we speculated that miR-21 could regulate the growth and development of HF cells through *KLF3* and thus affect the quality of wool. *TNPO1*, a translocation protein, facilitates the translocation of hnRNP A1 back to the nucleus. *TNPO1* can affect the expression of downstream genes in a variety of ways. The chemokine receptor CCR2 undergoes transportin1-dependent nuclear translocation (Favre et al., 2008). Additionally, CCR2 gene expression induces apoptosis and inhibits the proliferation, migration, and invasion of PC-3M cells (Gao et al., 2013). Therefore, it can be speculated that miR-21 affects cell growth and development through *TNPO1*-regulated apoptosis and proliferation via CCR2; HF cell proliferation and hair follicle development may be impacted through the miR-21-*TNPO1*-CCR2 pathway.

In summary, we found that overexpression or inhibition of miR-21 could affect the gene expression of *CNKSR2*, *KLF3* and *TNPO1*. Furthermore, the miR-21-*CNKSR2*-MAPK, miR-21-*KLF3*, miR-21-*TNPO1*-CCR2 pathway was found in HF cell development. Our results indicate a possible role for the involvement of miR-21 in HF development and suggest three potential targets genes of miR-21 that could be manually used to control HF development through gene therapy.

## Conclusions

Our work is the first study on the potential functions of miRNA-21 in hair follicle development.Here, we also found three new genes related to the miRNA-21 which were *CNKSR2, TNPO1* and *KLF3*. The miRNA-21 were negative correlation to three target gene in sheep with different wool quality. The data provide novel insights into the functions of miRNA-21 in hair follicle development.

##  Supplemental Information

10.7717/peerj.7167/supp-1 Dataset S1 Dual-luciferase fluorescence reports data Each data point indicates the fluorescence value.

10.7717/peerj.7167/supp-2 Dataset S2 RT-qPCR data between SM and STH Each data point indicates the Ct value.

10.7717/peerj.7167/supp-3 Dataset S3 RT-qPCR data between different months Each data point indicates the Ct value.

10.7717/peerj.7167/supp-4 Dataset S4 Construction of dual luciferase reporter vector The mutant vector and wild-type vector of the target gene were constructed by PCR, product purification, enzyme digestion, recycling, connection, transformation and colony PCR identification. Figures were PCR products, enzyme fragment and vector sequencing.

## Additional Information and Declarations

### Data Availability

The following information was supplied regarding data availability: Data is available in the Supplemental Files.
