# Supplementary material for: Identification of microRNA-21 target genes associated with hair follicle development in sheep
Source: PeerJ. 2019 Jun 27;7:e7167. doi: 10.7717/peerj.7167 (PMC6599667; doi:10.7717/peerj.7167)
Supplement: Dataset S4 — The mutant vector and wild-type vector of the target gene were constructed by PCR, product purification, enzyme digestion, recycling, connection, transformation and colony PCR identification. Figures were PCR products, enzyme fragment and vector sequencing. [file peerj-07-7167-s004.pdf]

双荧光素酶载体构建实验报告

实验简介

利用 PCR 方法，根据 KLF3 (Ovis aries) 3'UTR 序列信息设计其扩增引物，以 Ovis aries 基因组 DNA 为模板 PCR 扩增 KLF3 基因的3' UTR 序列，将其克隆到 **pmiR-RB-REPORT™**双荧光素酶报告载体中，所用载体的报告荧光为 hRluc，校正荧光为 hluc（做内参校正）。

实验步骤及结果：

一、 基因序列分析

对基因序列及载体序列分析，可以利用 XhoI，NotI 两个限制性内切酶将目的基因片段克隆到载体中，载体图谱如下：

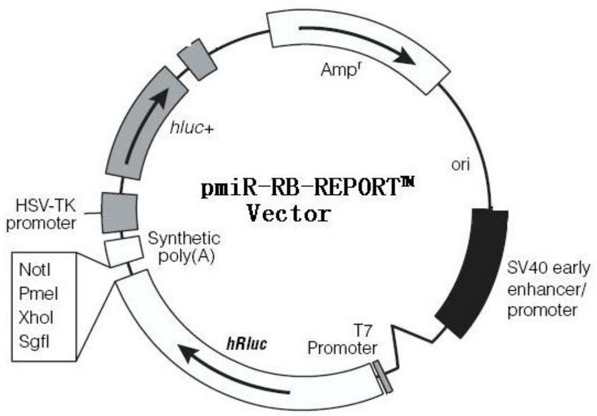

设计扩增引物如下：

oar_KLF3_3UTR_F: GGCGGCTCGAGAGTGTTGCACTAATGTGGA

oar_KLF3_3UTR_R: AATGCGGCCGCGAGTAGAAACAGAGAAGGAAC

红色分别为 XhoI，NotI 酶切位点序列，之前序列为保护碱基，引物扩增总长度为 607bp。

oar_KLF3_mut_F: TATATTTTTATTCGAAATAAGACTGAATGGGTA

oar_KLF3_mut_R: GTCTTATTTCGAATAAAAATATATTACACTTTA

二、 PCR

反应体系设计为30 μl 总体系，具体是5×Phusion Buffer 6 μl， 2.5 mM dNTP mix 2.4 μl，上下游引物（10 μM）各1 μl，Phusion DNA 聚合酶（2 U/μl）0.3 μl，DNA 模板1 μl（约100 ng），用灭菌水补足30 μl 体系。反应条件为（降落 PCR）：98℃ 2分钟预变性，循环内98℃ 10秒变性，从65℃每个循环降1℃退火，72℃延伸30秒，10个循环；再98℃ 10秒变性，60℃退火，72℃延伸30秒，进行15个循环；PCR 反应循环后72℃继续延伸3分钟，然后4℃保存。

PCR 反应完取5 μl PCR 产物进行1.5%琼脂糖电泳分析，如下：

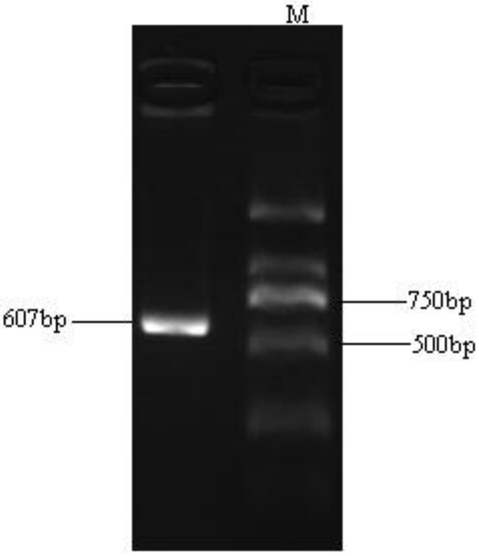

备注：M: D2000；扩增大小与理论大小相符。

三、PCR 产物的纯化

按照凝胶纯化试剂盒说明书进行纯化产物。

四、酶切

用 XhoI，NotI 对上述纯化产物和载体进行双酶切，酶切反应体系如下：10×H 缓冲液 4 μl，DNA 约2 μg，内切酶(10 U/μl) 各1 μl，灭菌去离子水补足到40 μl。反应条件37℃，4小时。

五、酶切回收纯化

酶切后回收纯化酶切产物。按照纯化试剂盒说明书进行纯化产物。

六、连接

连接反应体系如下：

目的片段 DNA 2 μl(约150 ng)，载体0.5 μl(约50 ng)，solution I 快速连接液5 μl, 灭菌去离子水补足到10 μl，16℃连接30分钟。

七、转化

1、取连接产物加到100 μl DH5α感受态细胞中混匀，冰浴30分钟。

2、将上述转化液置于42℃水浴90秒，取出后立即置于冰浴中放置2-3分钟。

3、向其中加入900 μl 37℃预热的LB（不含抗生素）培养基，150 rpm、37℃振荡培养45分钟。

4、2500 rpm离心5分钟，将上清液吸走，留100 μl混匀菌液，加到含Amp抗生素LB固体琼脂培养基上（抗生素浓度100 μg/ml），用无菌的弯头玻棒轻轻的将细胞均匀涂开。待平板表面干燥后，倒置平板，37℃培养12-16小时。

八、菌落 PCR 鉴定

挑取上述平板上的单菌落，溶到3 μl 水中，取0.5 μl 做模板，进行 PCR。反应体系设计为10 μl 总体系，具体是10×Taq buffer Buffer 1 μl，25 mM $MgCl_2$ 1.6 μl，2.5 mM dNTP mix 1 μl，载体上下游引物（10 μM）各0.5μl，Taq DNA 聚合酶（2.5 U/μl）0.3 μl，

DNA 模板0.5 µl （约100 ng），用灭菌水补足10 µl 体系。反应条件为95℃ 3分钟预变性，循环内95℃ 30秒变性，56℃退火，72℃延伸1分钟；20个循环； PCR 反应循环后72℃继续延伸3分钟，然后4℃保存。

实验共挑取5个菌落，进行 PCR。PCR 反应完取5 µl PCR 产物进行1.5%琼脂糖电泳分析，用载体上游和载体下游引物扩增的条带理论大小为850bp 左右，其实际扩增条带如下：

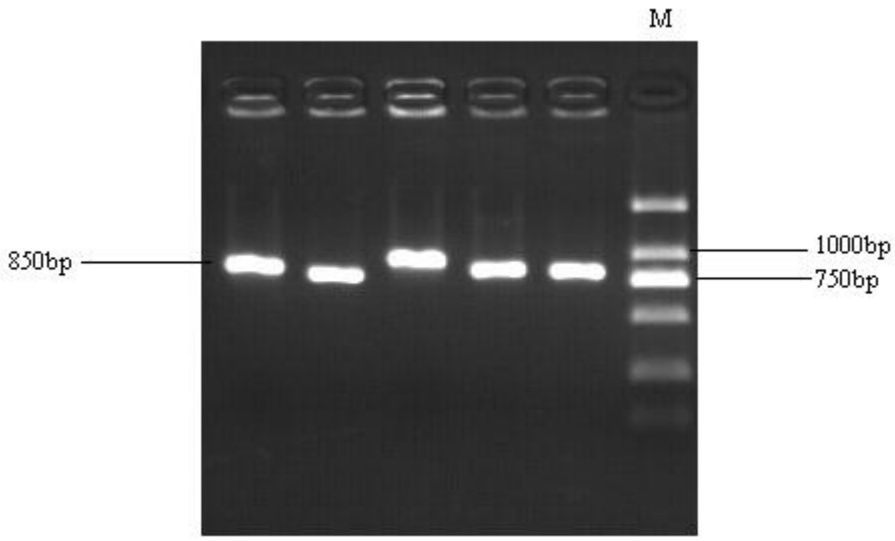

备注：M: D2000 ；菌 P 大小与理论大小相符。

九、将阳性的克隆送测序公司测序进一步鉴定（广州市锐博生物科技有限公司），详细测序结果请参考附件

(加黑划线标注为插入序列，两边为载体序列）

GGACGCTCCAGATGAAAATGGGTAAGTACATCAAGAGCTTCGTGGAGCGCGTGCTGAAGAACGAGCAGTAATTCTAGGCGA
TCGCTCGAGAGTGTTGCACTAATGTGGAATTTGAAAAACTATATTGCTGATACTGTATATCTGCACATCATTCCTGATTAGATT
GTTTAAAAGACAATCTCAAAGGTCTAAGGTTTTAAAATAATTTGGTTGGTTTGAAAATACACAGTCATCTTGAAGGAATCAC
TGTCATGCATGAAGTTGTTCAGAGTGGTCTTTATTCTCTTCTTGGTCAAGGTTAACAATTTCTAAATGATTGCAAATTCACTTA
AATAATACATTTACAAAGCCATTTTACATGCATTAAACGAGGGCTACAACAATACTATGTTTTACAAATACTAGCACTTTTTTT
TTGCTGTTATGTACTTAGTGTTAGAGGGTCAAAATAATCTTTCTGCTTAGCATCTCTTAAACCATACCTGCAAATATAGCAGGA
TTATTACATTTACAGTACTTTAATACTTGTATAAACTATGCAGAAATTTTTAATAAAGTGTAATATATTTTATAAGCTAATAAGAC
TGAATGGGTAAAGGTTTTTAGCATGCATTAGTATACTTGCAAATACTGAAACATTTTGGTAATCTTTCTTACTAAAGATGTGAA
TGTTTAATGTTCCTTCTCTGTTTCTACTCGCGGCCGCTGGCCGCAATAAAATATCTTTATTTTTCATTACATCTGTGTGTTGGTT
TTTTGTGTGAGGATCTAAATGAGTCTTCGGACCTCGCGGGGG

十、突变载体构建

利用 PCR 突变的方法，在野生型载体基础上，设计突变引物将靶序列 ATAAGCT 突变为 TATTCGA。

1、突变分段 PCR

以野生型载体为模板，分别扩增含突变序列的两段序列。反应体系设计为30 µl 总体系，具体是5×Phusion Buffer 6 µl， 2.5 mM dNTP mix 2.4 µl，上下游引物（10 µM）各1 µl（上游引物与突变下游引物配对，突变上游引物与下游引物配对），Phusion DNA 聚合酶（2 U/µl）0.3 µl，DNA 模板 1 µl（约100 ng），用灭菌水补足30 µl 体系。反应条件为（降落 PCR）：98℃ 2分钟预变性，循环内98℃ 10秒变性，从65℃每个循环降1℃退火，72℃延伸30秒；10个循环；再98℃ 10秒变性，60℃退火，72℃延伸30秒，进行15个循环；PCR 反应循环后72℃继续延伸3分钟，然后4℃保存。

PCR 反应完取 PCR 产物进行1.5%琼脂糖电泳分析并纯化回收。按照纯化试剂盒说明书进行纯化产物。

2、突变两段拼接

反应体系设计为10 µl 总体系，具体是上述纯化产物各取1 µl，5×Phusion Buffer 2 µl， 2.5 mM dNTP mix 0.8 µl。Phusion DNA 聚合酶（2 U/µl）0.1 µl ，用灭菌水补足10 µl 体系。反应条件为（降落 PCR）：98℃ 2分钟预变性，循环内98℃ 10 秒变性，从60℃每个循环降1℃退火，72℃延伸30秒，7个循环；再98℃ 10秒变性，55℃退火，72℃延伸30秒，进行10个循环；PCR 反应循环后72℃继续延伸3分钟，然后4℃保存。

3、拼接后 PCR

反应体系设计为30 µl 总体系，具体是5×Phusion Buffer 6 µl， 2.5 mM dNTP mix 2.4 µl，上下游引物（10 µM）各1 µl，Phusion DNA 聚合酶（2 U/µl）0.3 µl，DNA 模板1 µl（约100 ng），用灭菌水补足30 µl 体系。反应条件为（降落 PCR）：98℃ 2分钟预变性，循环内98℃ 10 秒变性，从65℃每个循环降1℃退火，72℃延伸30秒，10个循环；再98℃ 10秒变性，60℃退火，72℃延伸30秒，进行15个循环；PCR 反应循环后72℃继续延伸3分钟，然后4℃保存。

PCR 反应完取 PCR 产物进行1.5%琼脂糖电泳分析并纯化回收。按照纯化试剂盒说明书进行纯化产物。

4、酶切

用 XhoI，NotI 对上述纯化产物和载体进行双酶切，酶切反应体系如下:10×H 缓冲液 4 µl，DNA 约2 µg，内切酶(10 U/µl)各1 µl，灭菌去离子水补足到40 µl。反应条件37℃，4小时。

5、酶切回收纯化

酶切后回收纯化酶切产物。按照纯化试剂盒说明书进行纯化产物。

6、连接

连接反应体系如下：

目的片段 DNA 2 µl(约150ng)，载体0.5 µl(约50ng)，solution I 快速连接液 5 µl，灭菌去离子水补足到10 µl，16℃连接30分钟。

7、转化

1) 取连接产物10 µl 加到100 µl DH5α感受态细胞中，混匀，冰浴30分钟。

2) 将上述转化液置于42℃水浴90秒，取出后立即置于冰浴中放置2-3分钟。

3) 向其中加入900 µl 37℃预热的LB(不含抗生素)培养基，150 rpm、37℃振荡培养45分钟。

4) 2500 rpm离心5分钟，将上清液吸走，留100 µl混匀菌液，加到含**Amp**抗生素LB固体琼脂培养基上(抗生素浓度100 µg/ml)，用无菌的弯头玻棒轻轻的将细胞均匀涂开。待平板表面干燥后，倒置平板，37℃培养12-16小时。

8、菌落扩大培养提取质粒

挑取上述平板上的单菌落，接种到含 **Amp** 抗生素（抗生素浓度100 µg/ml）的4 ml LB 液体培养基中，于220 rpm 摇床培养12小时；然后提取质粒。

将质粒送测序公司测序鉴定，详细测序峰图结果请参考附件。

测序结果显示，成功将靶序列 ATAAGCT 突变为 TATTCGA。

地址：广州开发区科学城科学大道182号创新大厦C3区13层(510663) 电话：(020)32290075 传真：(020)32290137
13/F, Innovation Building C3, 182 kexue Avenue, Science Park, Guangzhou 510663, PR of China
免费热线：400 686 0075 企业QQ：4006860075 网站：www.sirna.cn www.ribobio.com 邮箱：support@ribobio.com

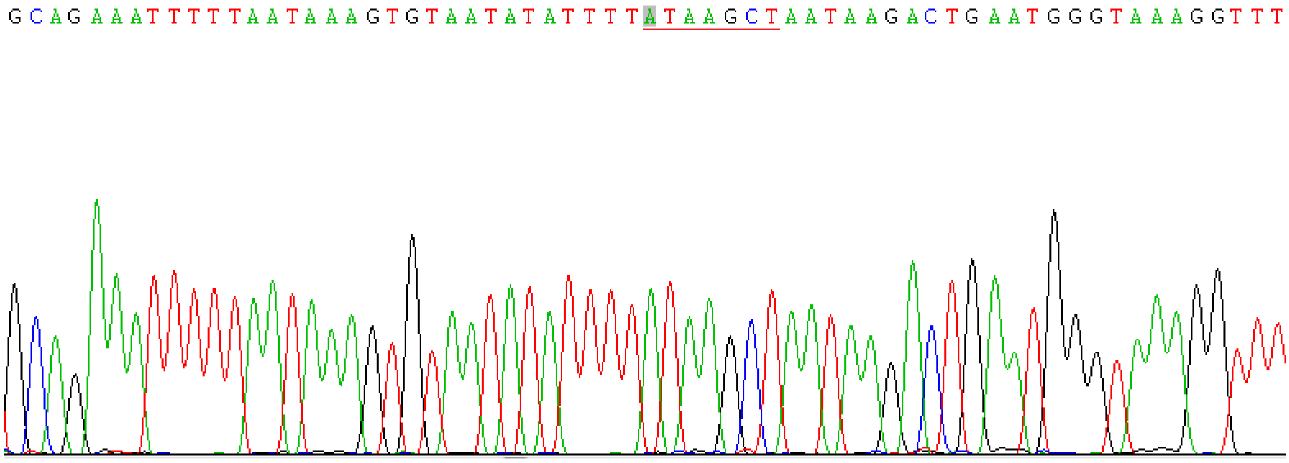

KLF3-WT

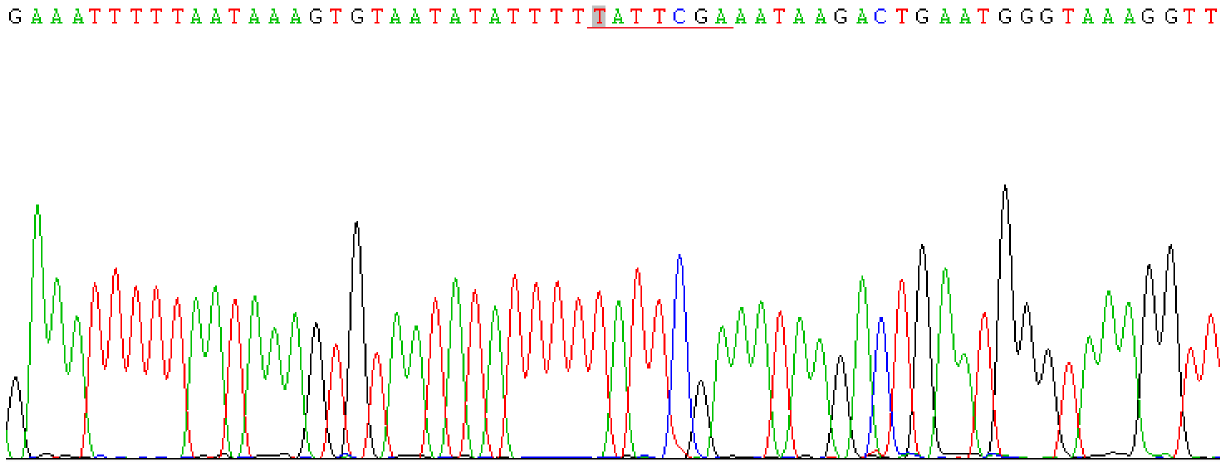

KLF3-MUT

主要试剂

LB 培养基：蛋白胨（tryptone），酵母提取物（yeast extract）	OXOID 公司（英）
限制性内切酶	Fermentas 公司
TIANprep Mini Plasmid Kit 质粒小提试剂盒	天根生化科技（北京）有限公司
Phusion DNA 聚合酶	Fermentas 公司
琼脂糖	西班牙 BioWest 公司
Universal DNA Purification Kit DNA 纯化回收.试剂盒	天根生化科技（北京）有限公司
连接反应液	Fermentas 公司
普通 DNA 聚合酶	Fermentas 公司
DH5α 感受态细胞	天根生化科技（北京）有限公司

主要仪器

PCR 仪器：　　Bio-RAD 公司

引物合成：　　苏州金唯智生物科技有限公司

序列测定：　　广州市锐博生物科技有限公司

如果有任何问题，请联系我们！

广州市锐博生物科技有限公司

地 址：广州开发区科学城科学大道 182 号创新大厦 C3 区 13 层

电 话：（020）32290075

邮 箱：service@ribobio.com

双荧光素酶载体构建实验报告

实验简介

利用 PCR 方法，根据 CNKSR2 (Ovis aries) 3'UTR 序列信息设计其扩增引物，以 Ovis aries 基因组 DNA 为模板 PCR 扩增 CNKSR2基因的3' UTR 序列，将其克隆到 **pmiR-RB-REPORT™**双荧光素酶报告载体中，所用载体的报告荧光为 hRluc，校正荧光为 hluc（做内参校正）。

实验步骤及结果：

一、 基因序列分析

对基因序列及载体序列分析，可以利用 XhoI，NotI 两个限制性内切酶将目的基因片段克隆到载体中，载体图谱如下：

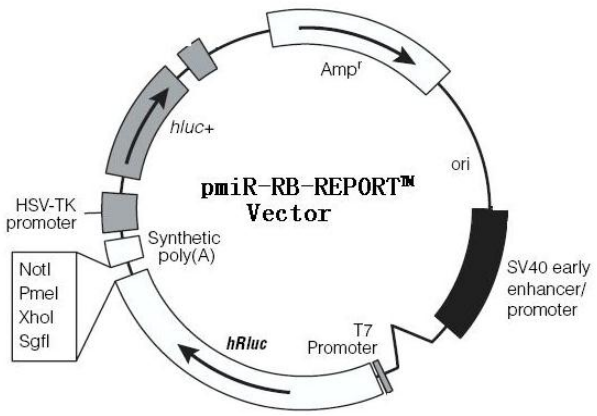

设计扩增引物如下：

oar_CNKSR2_3UTR_F: GGCGGCTCGAGTACACTGCGAGAGTTGGTAGA

oar_CNKSR2_3UTR_R: AATGCGGCCGCCAAGAAGTAAGGAGAAAGTTAGG

红色分别为 XhoI，NotI 酶切位点序列，之前序列为保护碱基，引物扩增总长度为 759bp。

oar_CNKSR2_mut_F: ACAGATTGTATTCGAAATGTTTAGAGAATTTAG

oar_CNKSR2_mut_R: TAAACATTTCGAATACAATCTGTGAGTCCAATG

二、 PCR

反应体系设计为30 μl 总体系，具体是5×Phusion Buffer 6 μl， 2.5 mM dNTP mix 2.4 μl，上下游引物（10 μM）各1 μl，Phusion DNA 聚合酶（2 U/μl）0.3 μl，DNA 模板1 μl（约100 ng），用灭菌水补足30 μl 体系。反应条件为（降落 PCR）：98℃ 2分钟预变性，循环内98℃ 10秒变性，从65℃每个循环降1℃退火，72℃延伸30秒，10个循环；再98℃ 10秒变性，60℃退火，72℃延伸30秒，进行15个循环；PCR 反应循环后72℃继续延伸3分钟，然后4℃保存。

PCR 反应完取5 μl PCR 产物进行1.5%琼脂糖电泳分析，如下：

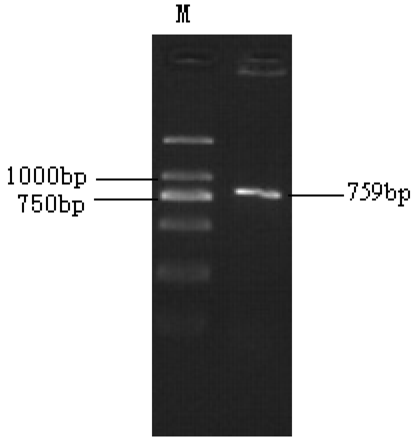

备注：M: D2000；扩增大小与理论大小相符。

三、PCR 产物的纯化

按照凝胶纯化试剂盒说明书进行纯化产物。

四、酶切

用 XhoI，NotI 对上述纯化产物和载体进行双酶切，酶切反应体系如下：10×H 缓冲液 4 μl，DNA 约2 μg，内切酶(10 U/μl) 各1 μl，灭菌去离子水补足到40 μl。反应条件37℃，4小时。

五、酶切回收纯化

酶切后回收纯化酶切产物。按照纯化试剂盒说明书进行纯化产物。

六、连接

连接反应体系如下：

目的片段 DNA 2 μl(约150 ng)，载体0.5 μl(约50 ng)，solution I 快速连接液5 μl, 灭菌去离子水补足到10 μl，16℃连接30分钟。

七、转化

1、取连接产物加到100 μl DH5α感受态细胞中混匀，冰浴30分钟。

2、将上述转化液置于42℃水浴90秒，取出后立即置于冰浴中放置2-3分钟。

3、向其中加入900 μl 37℃预热的LB（不含抗生素）培养基，150 rpm、37℃振荡培养45分钟。

4、2500 rpm离心5分钟，将上清液吸走，留100 μl混匀菌液，加到含Amp抗生素LB固体琼脂培养基上（抗生素浓度100 μg/ml），用无菌的弯头玻棒轻轻的将细胞均匀涂开。待平板表面干燥后，倒置平板，37℃培养12-16小时。

八、菌落 PCR 鉴定

挑取上述平板上的单菌落，溶到3 μl 水中，取0.5 μl 做模板，进行 PCR。反应体系设计为10 μl 总体系，具体是10×Taq buffer Buffer 1 μl，25 mM $MgCl_2$ 1.6 μl，2.5 mM dNTP mix 1 μl，载体上下游引物（10 μM）各0.5μl，Taq DNA 聚合酶（2.5 U/μl）0.3 μl，DNA 模板0.5 μl （约100 ng），用灭菌水补足10 μl 体系。反应条件为95℃ 3分钟预变性，循环内95℃ 30秒变性，56℃退火，72℃ 延伸1分钟；20个循环； PCR 反应循环后72℃继续延伸3分钟，然后4℃保存。

实验共挑取5个菌落，进行 PCR。PCR 反应完取5 μl PCR 产物进行1.5%琼脂糖电泳分析，用载体上游和载体下游引物扩增

的条带理论大小为1000bp 左右，其实际扩增条带如下：

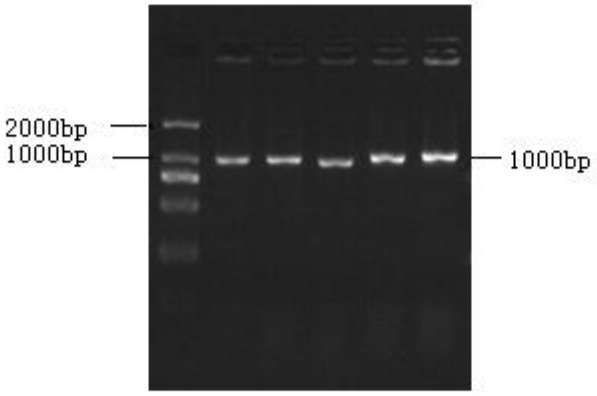

备注：M: D2000 ；菌 P 大小与理论大小相符。

九、 将阳性的克隆送测序公司测序进一步鉴定（广州市锐博生物科技有限公司），详细测序结果请参考附件

(加黑划线标注为插入序列，两边为载体序列）

ATGGGTAAGTACATCAAGAGCTTCGTGGAGCGCGTGCTGAAGAACGAGCAGTAATTCTAGGCGATCGCTCGAGTACACTGC
GAGAGTTGGTAGAACCTCTCCATGCCAAATCGGATCCACTTCTGTTGGCACTCAACCCATTGGACTCACAGATTGATAAGCT
AATGTTTAGAGAATTTAGATCGGAGAGAGTCGGCACGACGCAGACTTAACATCAACCTCTTGCAAGCAACTAAAATGGCCT
CGTCCTTGCTGTTTATAACAGAAAACAGACTTTTAAAAGCTTAGATCATCAAGTGTTTTGGATTGGGGGCCTCCCTAAAGGG
ATATTAAGAGGGGCAGGCCACTCTTAAGAAGAATGCAAGCTTTCTTCACTGGGACTAGCATAAGATCAAAGCCAATCAAGA
TGGAGCACAGTGACAGAAAACTGCTGTTTCTGTGGGTGAACAGAGGGAAAGGGCACTGACTGGGGAAGGGCTCAGTTGT
GTTCTCCTGTCACAGGAAAGGAGAGGGATCAGCTCCAATAACTAGAAAACCCTGGCTGTTCAATGGACTCTTAAGTGGCCT
CTTGAAGGCAAAGCAGAGAAAGCAAATTCTGTGTGTTAAGTGTATTTCGCATTTTTAAAATTTGACGTGCTGTATTGTACTAC
ATTAAGTGTAATCTTAAGGCAAGGTATCTACAACCTGCTCTGAAACTTACTATGTTTATTCTATTATAAAGTGTATTCAGGTGC
AACACAGAGACTGCTTTCAGTGGCACTAATGAAGAAAATGCCACATGCCAGGCTTTAGTAGATTCCTCAGCTAAAATCCTAA
CTTTCTCCTTACTTCTTGGCGGCCGCTGGCCGCAATAAAATATCTTTATTTCATTACATCTGT

十、 突变载体构建

利用 PCR 突变的方法，在野生型载体基础上，设计突变引物将靶序列 ATAAGCT 突变为 TATTCGA。

1、突变分段 PCR

以野生型载体为模板，分别扩增含突变序列的两段序列。反应体系设计为30 µl 总体系，具体是5×Phusion Buffer 6 µl， 2.5 mM dNTP mix 2.4 µl，上下游引物（10 µM）各1 µl（上游引物与突变下游引物配对，突变上游引物与下游引物配对），Phusion DNA 聚合酶（2 U/µl）0.3 µl，DNA 模板 1 µl（约100 ng），用灭菌水补足30 µl 体系。反应条件为（降落 PCR）：98℃ 2分钟预变性，循环内98℃ 10秒变性，从65℃每个循环降1℃退火，72℃延伸30秒；10个循环；再98℃ 10秒变性，60℃退火，72℃延伸30秒，进行15个循环；PCR 反应循环后72℃继续延伸3分钟，然后4℃保存。

PCR 反应完取 PCR 产物进行1.5%琼脂糖电泳分析并纯化回收。按照纯化试剂盒说明书进行纯化产物。

2、突变两段拼接

反应体系设计为10 µl 总体系，具体是上述纯化产物各取1 µl，5×Phusion Buffer 2 µl， 2.5 mM dNTP mix 0.8 µl。Phusion DNA 聚合酶（2 U/µl）0.1 µl ，用灭菌水补足10 µl 体系。反应条件为（降落 PCR）：98℃ 2分钟预变性，循环内98℃ 10 秒变性，从60℃每个循环降1℃退火，72℃延伸30秒，7个循环；再98℃ 10秒变性，55℃退火，72℃延伸30秒，进行10个循环；PCR 反应循

环后72℃继续延伸3分钟，然后4℃保存。

3、拼接后 PCR

反应体系设计为30 μl 总体系，具体是5×Phusion Buffer 6 μl， 2.5 mM dNTP mix 2.4 μl，上下游引物（10 μM）各1 μl，Phusion DNA 聚合酶（2 U/μl）0.3 μl，DNA 模板1 μl（约100 ng），用灭菌水补足30 μl 体系。反应条件为（降落 PCR）：98℃ 2分钟预变性，循环内98℃ 10 秒变性，从65℃每个循环降1℃退火，72℃延伸30秒，10个循环；再98℃ 10秒变性，60℃退火，72℃延伸30秒，进行15个循环；PCR 反应循环后72℃继续延伸3分钟，然后4℃保存。

PCR 反应完取 PCR 产物进行1.5%琼脂糖电泳分析并纯化回收。按照纯化试剂盒说明书进行纯化产物。

4、酶切

用 XhoI，NotI 对上述纯化产物和载体进行双酶切，酶切反应体系如下:10×H 缓冲液 4 μl，DNA 约2 μg，内切酶(10 U/μl)各1 μl，灭菌去离子水补足到40 μl。反应条件37℃，4小时。

5、酶切回收纯化

酶切后回收纯化酶切产物。按照纯化试剂盒说明书进行纯化产物。

6、连接

连接反应体系如下：

目的片段 DNA 2 μl(约150ng)，载体0.5 μl(约50ng)，solution I 快速连接液 5 μl，灭菌去离子水补足到10 μl，16℃连接30分钟。

7、转化

1) 取连接产物10 μl 加到100 μl DH5α感受态细胞中，混匀，冰浴30分钟。

2) 将上述转化液置于42℃水浴90秒，取出后立即置于冰浴中放置2-3分钟。

3) 向其中加入900 μl 37℃预热的LB(不含抗生素)培养基，150 rpm、37℃振荡培养45分钟。

4) 2500 rpm离心5分钟，将上清液吸走，留100 μl混匀菌液，加到含**Amp**抗生素LB固体琼脂培养基上（抗生素浓度100 μg/ml），用无菌的弯头玻棒轻轻的将细胞均匀涂开。待平板表面干燥后，倒置平板，37℃培养12-16小时。

8、菌落扩大培养提取质粒

挑取上述平板上的单菌落，接种到含 **Amp** 抗生素（抗生素浓度100 μg/ml）的4 ml LB 液体培养基中，于220 rpm 摇床培养12小时；然后提取质粒。

将质粒送测序公司测序鉴定，详细测序峰图结果请参考附件。

测序结果显示，成功将靶序列 ATAAGCT 突变为 TATTCGA。

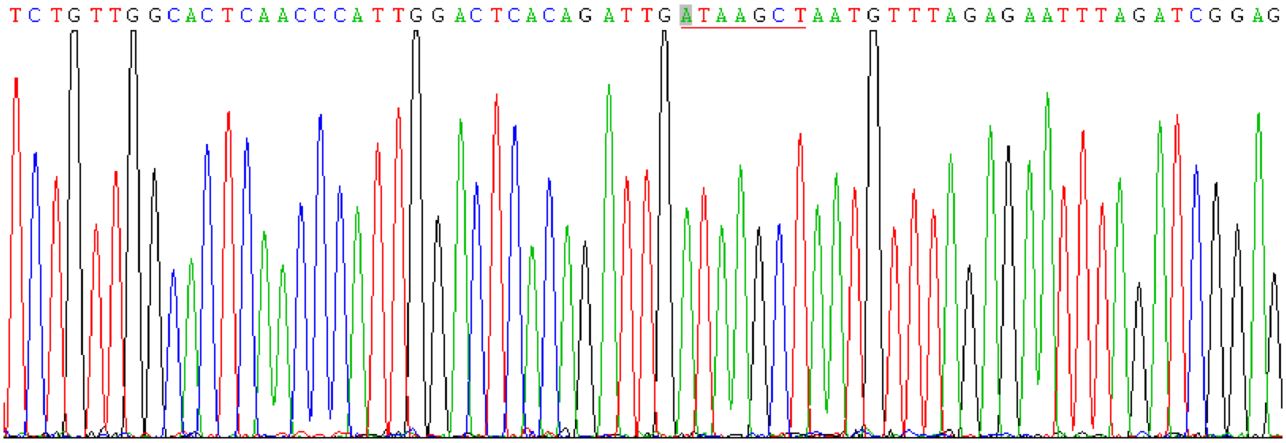

CNKSR2-WT

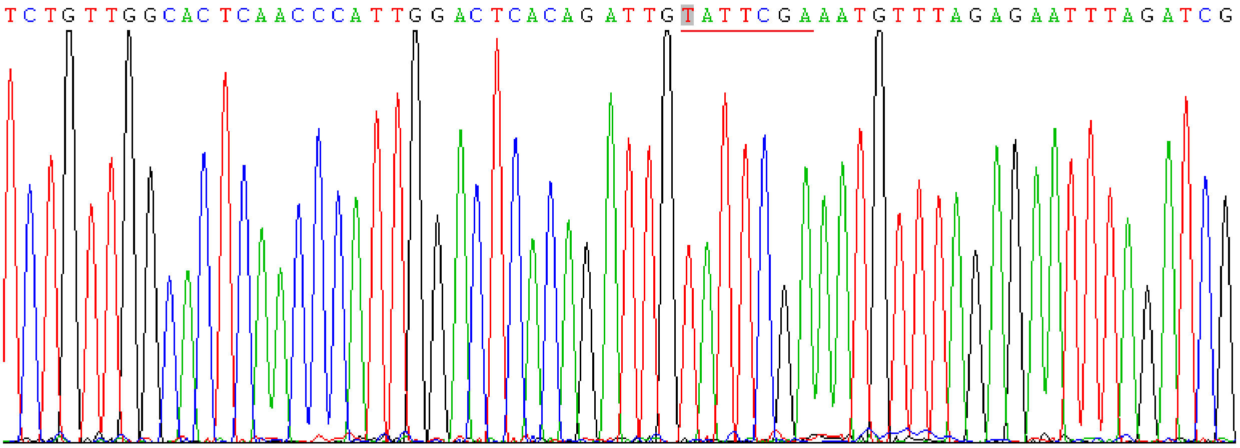

CNKSR2-MUT

主要试剂

LB 培养基：蛋白胨（tryptone），酵母提取物（yeast extract）	OXOID 公司（英）
限制性内切酶	Fermentas 公司
TIANprep Mini Plasmid Kit 质粒小提试剂盒	天根生化科技（北京）有限公司
Phusion DNA 聚合酶	Fermentas 公司
琼脂糖	西班牙 BioWest 公司
Universal DNA Purification Kit DNA 纯化回收.试剂盒	天根生化科技（北京）有限公司
连接反应液	Fermentas 公司
普通 DNA 聚合酶	Fermentas 公司
DH5α 感受态细胞	天根生化科技（北京）有限公司

主要仪器

广州市锐博生物科技有限公司
GUANGZHOU RIBOBIO CO., LTD
地址：广州开发区科学城科学大道182号创新大厦C3区13层(510663)　电话：(020)32290075　传真：(020)32290137
13/F, Innovation Building C3, 182 kexue Avenue, Science Park, Guangzhou 510663, PR of China
免费热线：400 686 0075　企业QQ：4006860075　网站：www.sirna.cn　www.ribobio.com　邮箱：support@ribobio.com

PCR 仪器：　　Bio-RAD 公司
引物合成：　　苏州金唯智生物科技有限公司
序列测定：　　广州市锐博生物科技有限公司

如果有任何问题，请联系我们！

广州市锐博生物科技有限公司
地 址：广州开发区科学城科学大道 182 号创新大厦 C3 区 13 层
电 话：（020）32290075
邮 箱：service@ribobio.com

双荧光素酶载体构建实验报告

实验简介

利用 PCR 方法，根据 KLF3 (Ovis aries) 3’UTR 序列信息设计其扩增引物，以 Ovis aries 基因组 DNA 为模板 PCR 扩增 KLF3 基因的3' UTR 序列，将其克隆到 **pmiR-RB-REPORT™**双荧光素酶报告载体中，所用载体的报告荧光为 hRluc，校正荧光为 hluc（做内参校正）。

实验步骤及结果：

一、 基因序列分析

对基因序列及载体序列分析，可以利用 XhoI，NotI 两个限制性内切酶将目的基因片段克隆到载体中，载体图谱如下：

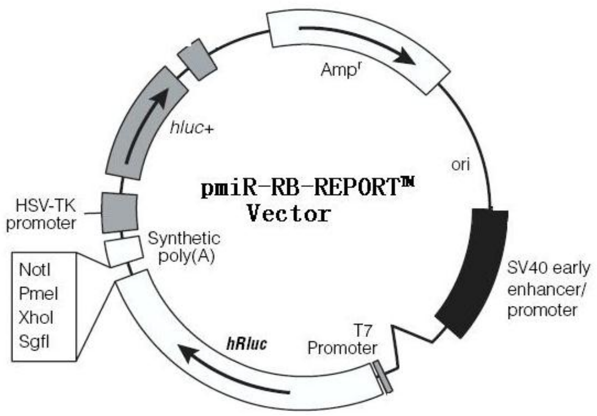

设计扩增引物如下：

oar_KLF3_3UTR_F: GGCGGCTCGAGAGTGTTGCACTAATGTGGA

oar_KLF3_3UTR_R: AATGCGGCCGCGAGTAGAAACAGAGAAGGAAC

红色分别为 XhoI，NotI 酶切位点序列，之前序列为保护碱基，引物扩增总长度为 607bp。

oar_KLF3_mut_F: TATATTTTTATTCGAAATAAGACTGAATGGGTA

oar_KLF3_mut_R: GTCTTATTTCGAATAAAAATATATTACACTTTA

二、 PCR

反应体系设计为30 µl 总体系，具体是5×Phusion Buffer 6 µl， 2.5 mM dNTP mix 2.4 µl，上下游引物（10 µM）各1 µl，Phusion DNA 聚合酶（2 U/µl）0.3 µl，DNA 模板1 µl（约100 ng），用灭菌水补足30 µl 体系。反应条件为（降落 PCR）：98℃ 2分钟预变性，循环内98℃ 10秒变性，从65℃每个循环降1℃退火，72℃延伸30秒，10个循环；再98℃ 10秒变性，60℃退火，72℃延伸30秒，进行15个循环；PCR 反应循环后72℃继续延伸3分钟，然后4℃保存。

PCR 反应完取5 µl PCR 产物进行1.5%琼脂糖电泳分析，如下：

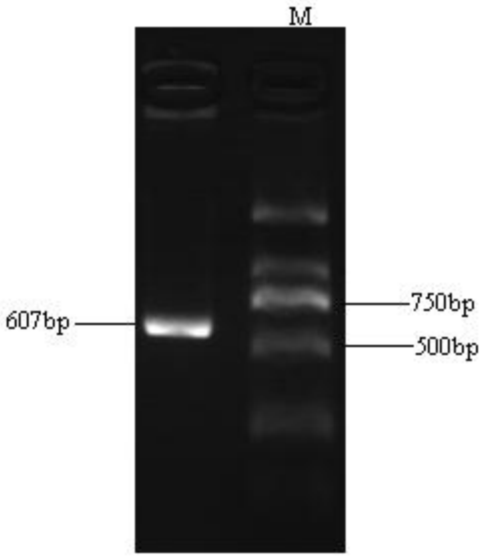

备注：M: D2000；扩增大小与理论大小相符。

三、PCR 产物的纯化

按照凝胶纯化试剂盒说明书进行纯化产物。

四、酶切

用 XhoI，NotI 对上述纯化产物和载体进行双酶切，酶切反应体系如下：10×H 缓冲液 4 μl，DNA 约2 μg，内切酶(10 U/μl)各1 μl，灭菌去离子水补足到40 μl。反应条件37℃，4小时。

五、酶切回收纯化

酶切后回收纯化酶切产物。按照纯化试剂盒说明书进行纯化产物。

六、连接

连接反应体系如下：

目的片段 DNA 2 μl(约150 ng)，载体0.5 μl(约50 ng)，solution I 快速连接液5 μl, 灭菌去离子水补足到10 μl，16℃连接30分钟。

七、转化

1、取连接产物加到100 μl DH5α感受态细胞中混匀，冰浴30分钟。

2、将上述转化液置于42℃水浴90秒，取出后立即置于冰浴中放置2-3分钟。

3、向其中加入900 μl 37℃预热的LB（不含抗生素）培养基，150 rpm、37℃振荡培养45分钟。

4、2500 rpm离心5分钟，将上清液吸走，留100 μl混匀菌液，加到含Amp抗生素LB固体琼脂培养基上（抗生素浓度100 μg/ml），用无菌的弯头玻棒轻轻的将细胞均匀涂开。待平板表面干燥后，倒置平板，37℃培养12-16小时。

八、菌落 PCR 鉴定

挑取上述平板上的单菌落，溶到3 μl 水中，取0.5 μl 做模板，进行 PCR。反应体系设计为10 μl 总体系，具体是10×Taq buffer Buffer 1 μl，25 mM $MgCl_2$ 1.6 μl，2.5 mM dNTP mix 1 μl，载体上下游引物（10 μM）各0.5μl，Taq DNA 聚合酶（2.5 U/μl）0.3 μl，

DNA 模板0.5 µl （约100 ng），用灭菌水补足10 µl 体系。反应条件为95℃ 3分钟预变性，循环内95℃ 30秒变性，56℃退火，72℃延伸1分钟；20个循环； PCR 反应循环后72℃继续延伸3分钟，然后4℃保存。

实验共挑取5个菌落，进行 PCR。PCR 反应完取5 µl PCR 产物进行1.5%琼脂糖电泳分析，用载体上游和载体下游引物扩增的条带理论大小为850bp 左右，其实际扩增条带如下：

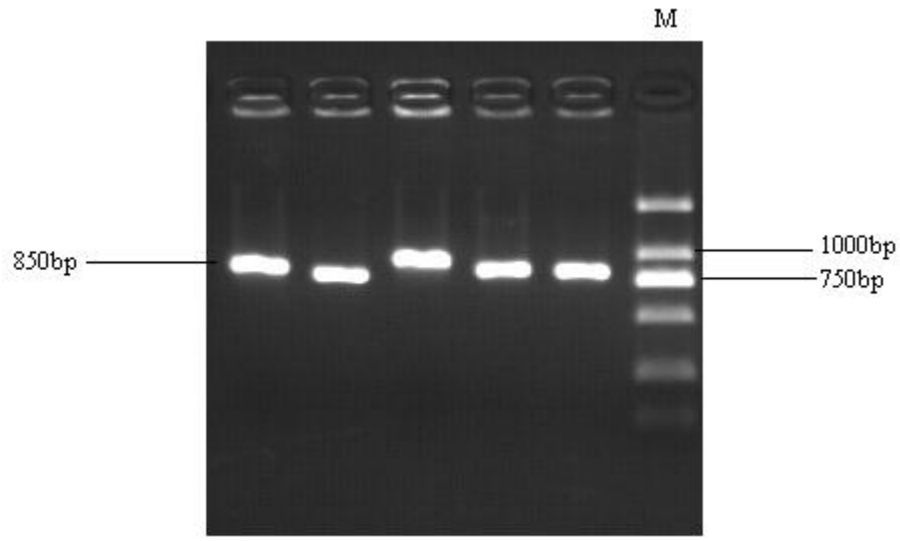

备注：M: D2000 ；菌 P 大小与理论大小相符。

九、将阳性的克隆送测序公司测序进一步鉴定（广州市锐博生物科技有限公司），详细测序结果请参考附件

(加黑划线标注为插入序列，两边为载体序列）

GGACGCTCCAGATGAAAATGGGTAAGTACATCAAGAGCTTCGTGGAGCGCGTGCTGAAGAACGAGCAGTAATTCTAGGCGA
TCGCTCGAGAGTGTTGCACTAATGTGGAATTTGAAAAACTATATTGCTGATACTGTATATCTGCACATCATTCCTGATTAGATT
GTTTAAAAGACAATCTCAAAGGTCTAAGGTTTTAAAATAATTTGGTTGGTTTGAAAATACACAGTCATCTTGAAGGAATCAC
TGTCATGCATGAAGTTGTTCAGAGTGGTCTTTATTCTCTTCTTGGTCAAGGTTAACAATTTCTAAATGATTGCAAATTCACTTA
AATAATACATTTACAAAGCCATTTTACATGCATTAAACGAGGGCTACAACAATACTATGTTTTACAAATACTAGCACTTTTTTT
TTGCTGTTATGTACTTAGTGTTAGAGGGTCAAAATAATCTTTCTGCTTAGCATCTCTTAAACCATACCTGCAAATATAGCAGGA
TTATTACATTTACAGTACTTTAATACTTGTATAAACTATGCAGAAATTTTTAATAAAGTGTAATATATTTTATAAGCTAATAAGAC
TGAATGGGTAAAGGTTTTTAGCATGCATTAGTATACTTGCAAATACTGAAACATTTTGGTAATCTTTCTTACTAAAGATGTGAA
TGTTTAATGTTCCTTCTCTGTTTCTACTCGCGGCCGCTGGCCGCAATAAAATATCTTTATTTTTCATTACATCTGTGTGTTGGTT
TTTTGTGTGAGGATCTAAATGAGTCTTCGGACCTCGCGGGGG

十、突变载体构建

利用 PCR 突变的方法，在野生型载体基础上，设计突变引物将靶序列 ATAAGCT 突变为 TATTCGA。

1、突变分段 PCR

以野生型载体为模板，分别扩增含突变序列的两段序列。反应体系设计为30 µl 总体系，具体是5×Phusion Buffer 6 µl， 2.5 mM dNTP mix 2.4 µl，上下游引物（10 µM）各1 µl（上游引物与突变下游引物配对，突变上游引物与下游引物配对），Phusion DNA 聚合酶（2 U/µl）0.3 µl，DNA 模板 1 µl（约100 ng），用灭菌水补足30 µl 体系。反应条件为（降落 PCR）：98℃ 2分钟预变性，循环内98℃ 10秒变性，从65℃每个循环降1℃退火，72℃延伸30秒；10个循环；再98℃ 10秒变性，60℃退火，72℃延伸30秒，进行15个循环；PCR 反应循环后72℃继续延伸3分钟，然后4℃保存。

PCR 反应完取 PCR 产物进行1.5%琼脂糖电泳分析并纯化回收。按照纯化试剂盒说明书进行纯化产物。

2、突变两段拼接

反应体系设计为10 μl 总体系，具体是上述纯化产物各取1 μl，5×Phusion Buffer 2 μl， 2.5 mM dNTP mix 0.8 μl。Phusion DNA 聚合酶（2 U/μl）0.1 μl ，用灭菌水补足10 μl 体系。反应条件为（降落 PCR）：98℃ 2分钟预变性，循环内98℃ 10 秒变性，从60℃每个循环降1℃退火，72℃延伸30秒，7个循环；再98℃ 10秒变性，55℃退火，72℃延伸30秒，进行10个循环；PCR 反应循环后72℃继续延伸3分钟，然后4℃保存。

3、拼接后 PCR

反应体系设计为30 μl 总体系，具体是5×Phusion Buffer 6 μl， 2.5 mM dNTP mix 2.4 μl，上下游引物（10 μM）各1 μl，Phusion DNA 聚合酶（2 U/μl）0.3 μl，DNA 模板1 μl（约100 ng），用灭菌水补足30 μl 体系。反应条件为（降落 PCR）：98℃ 2分钟预变性，循环内98℃ 10 秒变性，从65℃每个循环降1℃退火，72℃延伸30秒，10个循环；再98℃ 10秒变性，60℃退火，72℃延伸30秒，进行15个循环；PCR 反应循环后72℃继续延伸3分钟，然后4℃保存。

PCR 反应完取 PCR 产物进行1.5%琼脂糖电泳分析并纯化回收。按照纯化试剂盒说明书进行纯化产物。

4、酶切

用 XhoI，NotI 对上述纯化产物和载体进行双酶切，酶切反应体系如下:10×H 缓冲液 4 μl，DNA 约2 μg，内切酶(10 U/μl)各1 μl，灭菌去离子水补足到40 μl。反应条件37℃，4小时。

5、酶切回收纯化

酶切后回收纯化酶切产物。按照纯化试剂盒说明书进行纯化产物。

6、连接

连接反应体系如下：

目的片段 DNA 2 μl(约150ng)，载体0.5 μl(约50ng)，solution I 快速连接液 5 μl，灭菌去离子水补足到10 μl，16℃连接30分钟。

7、转化

1) 取连接产物10 μl 加到100 μl DH5α感受态细胞中，混匀，冰浴30分钟。

2) 将上述转化液置于42℃水浴90秒，取出后立即置于冰浴中放置2-3分钟。

3) 向其中加入900 μl 37℃预热的LB(不含抗生素)培养基，150 rpm、37℃振荡培养45分钟。

4) 2500 rpm离心5分钟，将上清液吸走，留100 μl混匀菌液，加到含**Amp**抗生素LB固体琼脂培养基上（抗生素浓度100 μg/ml），用无菌的弯头玻棒轻轻的将细胞均匀涂开。待平板表面干燥后，倒置平板，37℃培养12-16小时。

8、菌落扩大培养提取质粒

挑取上述平板上的单菌落，接种到含 **Amp** 抗生素（抗生素浓度100 μg/ml）的4 ml LB 液体培养基中，于220 rpm 摇床培养12小时；然后提取质粒。

将质粒送测序公司测序鉴定，详细测序峰图结果请参考附件。

测序结果显示，成功将靶序列 ATAAGCT 突变为 TATTCGA。

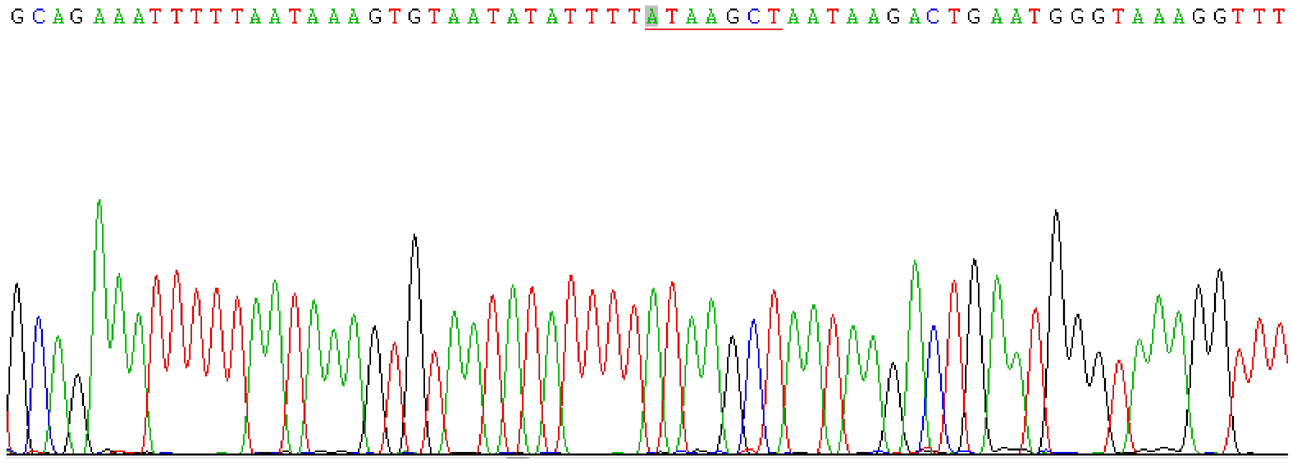

KLF3-WT

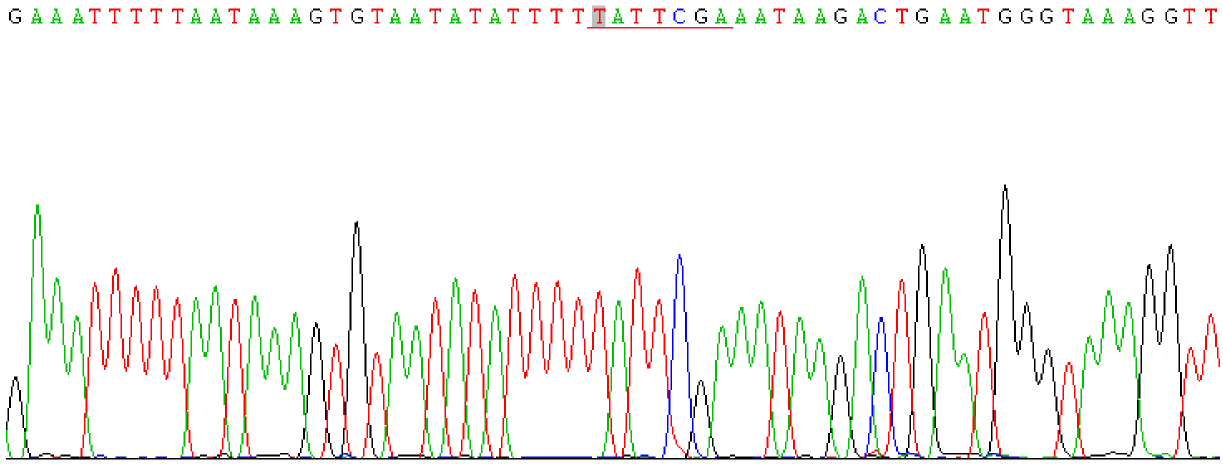

KLF3-MUT

主要试剂

LB 培养基：蛋白胨（tryptone），酵母提取物（yeast extract）	OXOID 公司（英）
限制性内切酶	Fermentas 公司
TIANprep Mini Plasmid Kit 质粒小提试剂盒	天根生化科技（北京）有限公司
Phusion DNA 聚合酶	Fermentas 公司
琼脂糖	西班牙 BioWest 公司
Universal DNA Purification Kit DNA 纯化回收.试剂盒	天根生化科技（北京）有限公司
连接反应液	Fermentas 公司
普通 DNA 聚合酶	Fermentas 公司
DH5α 感受态细胞	天根生化科技（北京）有限公司

广州市锐博生物科技有限公司
GUANGZHOU RIBOBIO CO., LTD
地址：广州开发区科学城科学大道182号创新大厦C3区13层(510663)　电话：(020)32290075　传真：(020)32290137
13/F, Innovation Building C3, 182 kexue Avenue, Science Park, Guangzhou 510663, PR of China
免费热线：400 686 0075　企业QQ：4006860075　网站：www.sima.cn　www.ribobio.com　邮箱：support@ribobio.com

主要仪器

PCR 仪器：　　Bio-RAD 公司

引物合成：　　苏州金唯智生物科技有限公司

序列测定：　　广州市锐博生物科技有限公司

如果有任何问题，请联系我们！

广州市锐博生物科技有限公司
地　址：广州开发区科学城科学大道 182 号创新大厦 C3 区 13 层
电　话：（020）32290075
邮　箱：service@ribobio.com
